# WxS-QC—a quality control pipeline for human germline short-variant Whole-Genome and Whole-Exome cohorts for population-scale analyses

**DOI:** 10.1093/bioinformatics/btag527

**Published:** 2026-07-20

**Authors:** Gennadii Zakharov, Ruth Eberhardt, Alina Frolova, Dmytro Horyslavets, Eric Hidari, Iaroslav Popov, Joanna Dennehy, Mykhailo Koreshkov, Pavlos Antoniou, Valeriia Kantsypa, Vladimir Ovchinnikov, Vivek Iyer

**Affiliations:** Wellcome Sanger Institute (WSI), Hinxton, Cambridgeshire CB10 1SA, United Kingdom; Wellcome Sanger Institute (WSI), Hinxton, Cambridgeshire CB10 1SA, United Kingdom; Institute of Molecular Biology and Genetics of NASU (IMBG), Kyiv 03143, Ukraine; Kyiv Academic University, 36 Akademika Vernads’koho Blvd, Kyiv 03142, Ukraine; Institute of Molecular Biology and Genetics of NASU (IMBG), Kyiv 03143, Ukraine; Kyiv Academic University, 36 Akademika Vernads’koho Blvd, Kyiv 03142, Ukraine; Wellcome Sanger Institute (WSI), Hinxton, Cambridgeshire CB10 1SA, United Kingdom; Wellcome Sanger Institute (WSI), Hinxton, Cambridgeshire CB10 1SA, United Kingdom; Wellcome Sanger Institute (WSI), Hinxton, Cambridgeshire CB10 1SA, United Kingdom; Institute of Molecular Biology and Genetics of NASU (IMBG), Kyiv 03143, Ukraine; Kyiv Academic University, 36 Akademika Vernads’koho Blvd, Kyiv 03142, Ukraine; Mars, Hansa Road, Hardwick Industrial Estate, Kings Lynn PE30 4JE, United Kingdom; Institute of Molecular Biology and Genetics of NASU (IMBG), Kyiv 03143, Ukraine; Educational and Scientific Institute of High Technologies, Taras Shevchenko National University of Kyiv, Kyiv 03022, Ukraine; Wellcome Sanger Institute (WSI), Hinxton, Cambridgeshire CB10 1SA, United Kingdom; Wellcome Sanger Institute (WSI), Hinxton, Cambridgeshire CB10 1SA, United Kingdom

## Abstract

**Summary:**

Whole-exome (WES) and whole-genome (WGS) sequencing are rapidly becoming preferred methods for population-scale analysis of the human genetic landscape. However, there are currently no standardized quality control (QC) pipelines for human WES and WGS datasets. In this paper, we present WxS-QC, a powerful, scalable, and convenient pipeline for the QC of human germline short-variant WGS and WES cohorts for population-scale analyses. Our pipeline is suitable for both rare-variant discovery and common-variant association studies. It is based on deeply refactored gnomAD v3 and v4 quality control pipelines, contains several methods we have developed de novo, and is aligned with current best practices in WGS/WES germline cohort QC. We provide all methods in a single codebase, aligned to work together and controlled via a single YAML config, with automatic export of resulting graphs and summary tables, excellent performance and scalability, and comprehensive documentation. The pipeline can run in any UNIX-like environment and can efficiently process cohorts of up to 200 000 whole-exome samples, with the potential to handle bigger datasets.

**Availability and implementation:**

The pipeline code is written in Python using the Hail library and is freely available under the BSD-3 license here: https://github.com/wtsi-hgi/wxs-qc. The detailed description of the pipeline is available in the pipeline documentation: https://github.com/wtsi-hgi/wxs-qc/blob/main/README.md. We also provide an open dataset with all required metadata, which is available at https://wxs-qc-data.cog.sanger.ac.uk/wxs-qc_public_dataset_v3.tar. An example of test dataset analysis is available in the supplementary materials.

## 1 Introduction

The advent of high-throughput sequencing technologies has revolutionized biological research and clinical diagnostics. Whole-exome sequencing (WES) and whole-genome sequencing (WGS) are rapidly becoming preferred methods for population-scale association studies of complex traits (Sealock *et al.* 2025). Efforts focusing on collecting and harmonizing sequencing data range from large cohorts such as the Genome Aggregation Database (Chen *et al.* 2024) and UK BioBank (Van Hout *et al.* 2020) to smaller disease-specific or ethnically specialized cohorts (Deciphering Developmental Disorders Study 2015, Sazonovs *et al.* 2022, Koko *et al.* 2024, Kim *et al.* 2026).

Prior to being subjected to statistical analysis, every cohort requires a careful quality control process to remove poor-quality samples and to distinguish true variants from technical artefacts (Sealock *et al.* 2025). Despite the rapid development of software tools for genome data analysis, there is currently no standardized WES and WGS data quality filtering pipeline for the purpose of population-scale association analysis (Sealock *et al.* 2025). The GA4GH WGS QC Standards initiative (https://www.ga4gh.org/product/wgs-quality-control-standards/) provides alignment-level and variant-level QC metrics (https://github.com/ga4gh/quality-control-wgs), but it doesn’t specify an exact approach for defining filtering thresholds.

One of the recent attempts to fill this gap is the tutorial (Sealock *et al.* 2025), which provides guidelines for conducting QC of samples and variants, and compares commonly used software programs for quality control. The tutorial authors suggest using the Hail library (Poterba *et al.* 2025) due to its scalability and flexibility, to provide a simple pipeline for genome/exome data QC. However, this pipeline uses hardcoded paths and constants for configuration, and provides no visualizations or export of results. Also, it does not have a variant QC module and relies on external GATK VQSR scores (Auwera and O’Connor 2020). It should be noted that this limitation has been overcome when using more recent ML-based variant callers, such as DeepVariant (Poplin *et al.* 2018), which provides more robust results (Barbitoff *et al.* 2022). The recently published DRAGEN pipeline (Behera *et al.* 2025) also relies on the variant caller quality and does not require extensive variant QC.

A more powerful and comprehensive solution is provided by the codebase used for data QC of the gnomAD database (Chen *et al.* 2024). This code generally aligns with the tutorial (Sealock *et al.* 2025) for sample QC. Version 3 also uses the Random Forest algorithm for variant QC and is not dependent on external variant QC solutions. However, this codebase is tightly coupled to the Broad Institute infrastructure and data, which makes it challenging to use outside the Broad Institute.

As a result, there is currently no open solution for a powerful, scalable, and convenient QC solution for human genome/exome germline short-variant WGS and WES cohorts for population-scale analyses.

## 2 Results and discussion

In this paper, we present *WxS-QC*, the pipeline for QC of human germline short-variant (SNVs and short indels) cohorts for population-scale analysis, including both rare variant discovery and common variants association studies. It aligns many well-tested QC approaches and unifies them into a single, configurable, infrastructure-agnostic solution.

### 2.1 Pipeline scheme

**Figure 1:**
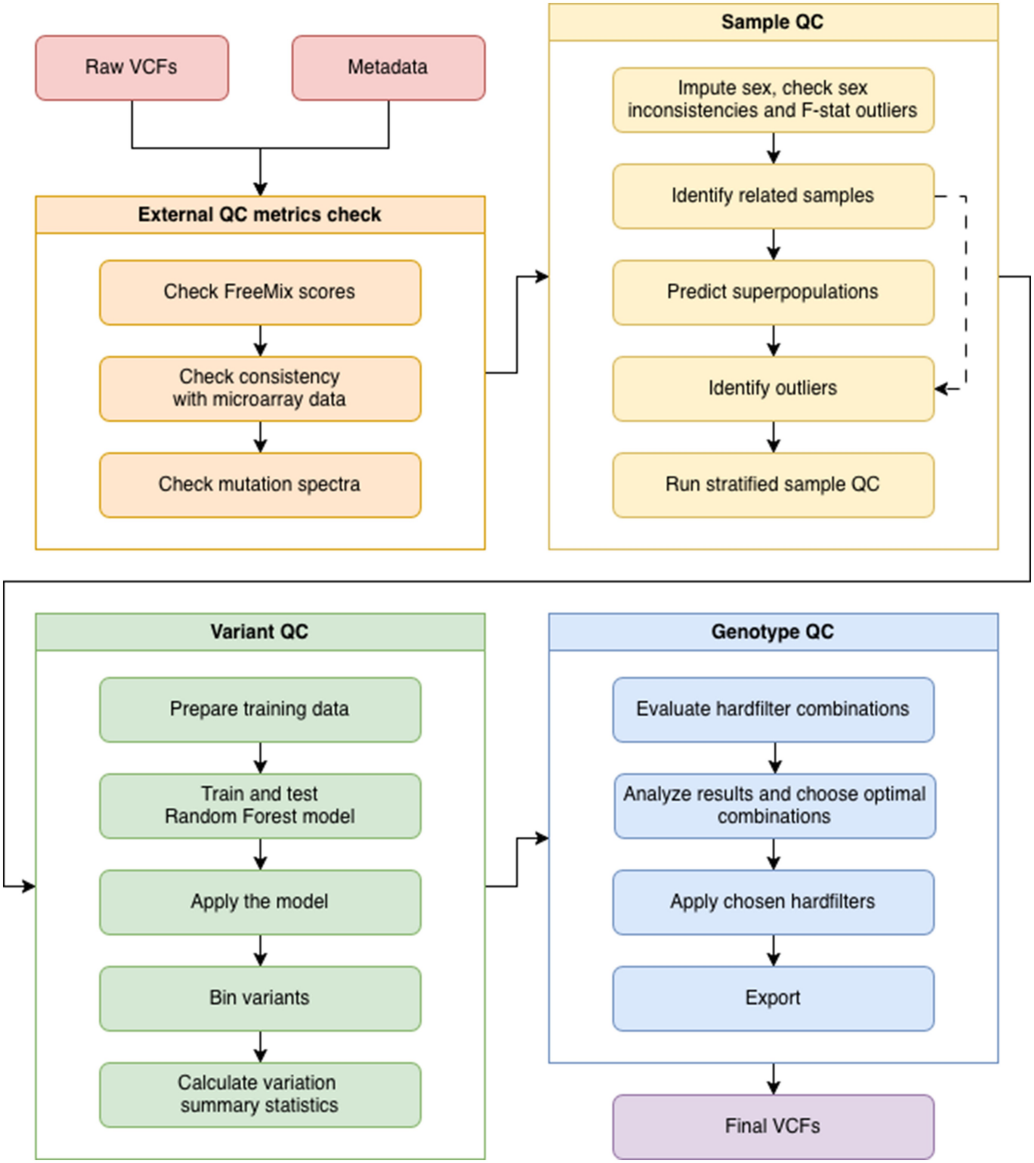
The high-level scheme of the WxS QC pipeline.

The high-level pipeline schema is shown in Fig. 1.

1. We tailor the pipeline to variants called with GATK, but you can use results from any “classic” variant caller, producing a rich set of variant-level statistics. The adaptation of the variant QC for modern neural network-based callers, like DeepVariant (Poplin *et al*. 2018) and DRAGEN (Behera *et al*. 2025), is underway.
  The pipeline also accepts optional metadata: contamination check provided by VerifyBamID2 (Zhang, Flickinger *et al.* 2020), self-reported sex, pedigree information, etc. The complete set of metadata is described in the pipeline documentation: https://github.com/wtsi-hgi/wxs-qc/blob/main/docs/wxs-qc_howto.md. The pipeline validates samples against the provided metadata and reports inconsistencies.
2. The sample QC step generally follows the approach described in (Sealock *et al*. 2025): calculate sample-level statistics (like number of SNVs/indels, …), choose a reference group for each sample, identify outliers, and remove them. For details, please refer to the pipeline howto: https://github.com/wtsi-hgi/wxs-qc/blob/main/docs/wxs-qc_howto.md#stage-2-sample-qc. To define a reference group for each sample in heterogeneous datasets, we implemented several approaches:
  - Assign a superpopulation to each sample and compare samples within superpopulations. We do it using principal component analysis (PCA) (Patterson *et al.* 2006, Reich *et al.* 2008) on the data from the 1000 Genomes study (1000 Genomes Project Consortium *et al*. 2015), projecting PCA scores onto the samples from the study to predict superpopulations, and applying the superpopulation-stratified threshold to filter samples. This approach makes PC axes more stable and not distorted by related samples or unusual ancestry (Wang *et al*. 2015, Zhang, Dey *et al.* 2020). As a result, we can handle datasets with any number of related individuals, and PCs from different studies remain visually comparable.
  - Since the PCA method can produce false outliers for samples whose ancestries are not represented in the 1000 Genomes data (which is common for non-European populations), we ported from gnomADv4 nearest-neighbour and linear regression QC approaches, which can handle complex datasets with mixed ancestries. More details are available in the pipeline howto: https://github.com/wtsi-hgi/wxs-qc/blob/main/docs/wxs-qc_howto.md#stage-2-sample-qc.
3. The variant QC step uses the approach from gnomAD v2 (Karczewski *et al*. 2020) and the first stages of gnomAD v3. We use a set of open resources to label variations as likely True-Positives (TP) and likely False-Positives (FP). Then we train a random forest (RF) model to predict TP and FP variants based on variant-level statistics, and use RF model score to group variants into several bins based on their reliability. The detailed description of the Variant QC is described in the pipeline how-to (https://github.com/wtsi-hgi/wxs-qc/blob/main/docs/wxs-qc_howto.md#stage-3-variant-qc) and supplementary materials, available as supplementary data at *Bioinformatics* online. Historically, this step was designed for GATK, which requires recalibrating the call correctness. In the development version, we’re updating this part to support DeepVariant (Poplin *et al*. 2018) and other neural network-based callers, which provide a reduced set of variant-level covariates.
4. In the final module, hard filter evaluation and genotype QC, we test how different combinations of RF model score and genotype-level filters affect the total resulting genotypes and variants. For each combination, we calculate TP/FP percentages, precision, and recall, allowing scientists to estimate the specificity and sensitivity of the filter. The detailed description of this step is available in the pipeline documentation and in the paper (Koko *et al*. 2024).

### 2.2 Key advantages of the pipeline

The main advantage of our pipeline is the way we provide all the methods to pipeline users. We provide:

- Well-tested methods from gnomAD v3 and v4 (Chen *et al*. 2024):
- Novel QC techniques that were developed while working with human germline WES and WGS cohorts (Koko *et al*. 2024, Kim *et al*. 2026). In particular:
  - The PCA projection approach makes superpopulation prediction based on a reference panel more robust.
  - The “genotype hardfilter evaluation” step allows the user to estimate the effect of different filter threshold combinations. Users can choose an optimal combination of variant and genotype-level filters to suit their scientific goal.
- All in one repo, designed to work together and aligned with best practices described in (Sealock *et al*. 2025).
- Easily configurable via a YAML-based config file.
- Infrastructure-agnostic solution that can run in any environment supported by the Hail library: a local machine, an on-premises Spark cluster, or a commercial cloud.
- Automatic export of summary tables and interactive graphs.
- A comprehensive, user-friendly tutorial describing the analysis process along with an open test dataset. The complete example of the QC for the public test data is described in the supplementary materials, available as supplementary data at *Bioinformatics* online.
- Excellent scalability due to the usage of the Hail library (https://github.com/hail-is/hail) (Poterba *et al*. 2025). The pipeline has been tested on cohorts of 170 000 whole-exome samples and 770 whole-genome samples.
- A reproducible solution that can handle cohorts for different types of experiments in a uniform manner.

### 2.3 Pipeline limitations

- Short-variants: SNVs and small indels. No structural variants support.
- Tested only on Illumina short-read data. Technically, there are no restrictions on running the pipeline for long-read data, as long as it provides a standard multi-sample VCF file as input.
- All sample QC approaches are designed for population-scale studies. We recommend having >=120 independent samples (200+ per ancestry/stratum is preferable) to obtain reliable results.
- We have tested the variant QC extensively, using only data produced by GATK v4 (Auwera and O’Connor 2020). However, by altering the inputs to the RF model, the variant QC could be adapted for other variant callers (FreeBayes (Garrison and Marth 2012), Strelka2 (Kim *et al*. 2018), etc).

## 3 Conclusion

The WxS-QC pipeline is a comprehensive tool that retains the power of the original gnomAD QC pipeline while adding novel QC approaches, documentation, visualizations, open test data, and infrastructure-agnostic operation. We believe that this pipeline is helpful for all scientists who run population-scale analysis of human cohorts of germline short variations.

In the supplementary materials, available as supplementary data at *Bioinformatics* online, we demonstrate QC analysis for the example dataset, analyze and discuss the results. We show that the WxS-QC pipeline achieves higher precision levels than standard threshold-based filtering, with comparable recall levels, particularly for indels.

## Supplementary Material

btag527_Supplementary_Data

## Data Availability

The data underlying this article are available in the GitHub repository https://github.com/wtsi-hgi/wxs-qc and on the Wellcome Sanger Institute data portal and can be accessed via the following links: The pipeline code and documentation are freely available via the WSI GitHub repository: https://github.com/wtsi-hgi/wxs-qc The resource bundle (8 Gb) is available to download from https://wxs-qc-data.cog.sanger.ac.uk/wxs-qc_resources.tar The open test dataset (300 Mb) is available to download from https://wxs-qc-data.cog.sanger.ac.uk/wxs-qc_public_dataset_v3.tar The code revision 0.9.2 used for the analysis described in the supplementary materials, available as supplementary data at *Bioinformatics* online is published here: https://doi.org/10.5281/zenodo.20397236
